# Sore Throat to Still’s: Group A Streptococcus Causing Adult-Onset Still’s Disease

**DOI:** 10.7759/cureus.69201

**Published:** 2024-09-11

**Authors:** Mandeep Kaur, Samantha W.S. Lo, Yixin Liu, Kevin Yip

**Affiliations:** 1 Internal Medicine, Wyckoff Heights Medical Center, New York City, USA; 2 Internal Medicine, Icahn School of Medicine at Mount Sinai Elmhurst Hospital Center, New York City, USA; 3 Social Services, Mount Sinai Health System, New York City, USA; 4 Rheumatology, Wyckoff Heights Medical Center, New York City, USA

**Keywords:** adult-onset acute rheumatic fever, adult-onset still’s disease, autoinflammatory disease, group a β hemolytic streptococci, hyperferritinemia

## Abstract

Adult-onset Still's disease (AOSD) is a rare hyper-inflammatory disease with poorly understood etiology, often presenting with nonspecific symptoms such as fever, inflammatory polyarthralgia, transient salmon-pink maculopapular rash, lymphadenopathy, and hepatosplenomegaly. We are presenting an unusual case of AOSD triggered by Group A streptococci (GAS) throat infection. We report the case of a 37-year-old male with no significant medical history admitted to medicine service after three emergency room (ER) visits. Our patient had a confirmed GAS throat infection and initially met the Jones criteria. However, further testing revealed significantly high inflammatory markers, clinically evident symmetrical synovitis in wrists and left knee, and widespread lymphadenopathy with worsening maculopapular rash. Given this, he met the Yamaguchi criteria, resulting in a diagnosis of AOSD. Group A Streptococcus infections are usually linked to acute rheumatic fever (ARF), but in our case, we believe GAS infection triggered the cascade of inflammatory responses resulting in AOSD. The patient received treatment with non-steroidal anti-inflammatory drugs (NSAIDs) and high-dose steroids with a resolution of arthralgia, down-trending ferritin, and clinical improvement in a skin rash. To the best of our knowledge, we are reporting the first case of GAS-triggered AOSD, highlighting the need to uncover atypical causes for AOSD and warranting the need to investigate triggers for AOSD.

## Introduction

Adult-onset Still's disease (AOSD) is an uncommon systemic autoimmune disorder with poorly understood etiology and often is a diagnosis of exclusion that contributes to delay in diagnosis, so it is crucial to have a clear diagnostic flow [1]. Adult-onset Still's disease has a rather heterogeneous presentation; however, it commonly manifests as fever of unknown origin, inflammatory polyarthralgia, transient salmon-pink maculopapular rash, lymphadenopathy, and hepatosplenomegaly [2]. Case reports have identified various triggers of AOSD, including the COVID-19 vaccine, bacterial and viral infections, other autoimmune diseases, and trauma [3]. To our knowledge, this is the first clinical case of Group A streptococci (GAS)-triggered AOSD. There is no specific diagnostic test for AOSD; some common laboratory findings are elevated inflammatory markers, including erythrocyte sedimentation rate (ESR), C-reactive protein (CRP), ferritin, thrombocytosis, above-normal aspartate aminotransferase (AST) and alanine aminotransferase (ALT), and anemia [4]. Adult-onset Still's disease has an annual incidence of 0.1 to 0.4 cases per 100,000 population, with a higher incidence in females and a bimodal age distribution between the ages of 15-25 years and 36-46 years [5]. Adult-onset Still's disease is hypothesized as a reactive disease occurring in predisposed individuals after exposure to different triggers like infections causing immune dysregulation. Inflammatory cytokines like interleukin (IL)-18 and IL-1 activate neutrophils and macrophages, and on the other hand, helper T cells, predominantly Th-17-related cytokines, are implicated in the pathogenesis of AOSD [5, 6]. Interleukin-18 is one of the most critical factors associated with disease activity and may help in diagnosis [7], while IL-1β is another marker linked to the etiopathogenesis of AOSD [8]. Interleukin-2 levels have been correlated to disease activity and found to be higher in the acute phase, reflecting that it can be used to monitor the activity of the disease [9].

## Case presentation

A 37-year-old Ecuadorian male with no known medical history was initially evaluated in the ER on day three after his symptoms started for a pruritic rash on his upper back and left lower abdomen. During the first ER visit, there were concerns about shingles or insect bites; the patient was discharged from the ED after a dose of diphenhydramine with hydrocortisone 1% topical cream because of a localized allergic reaction. The patient had an insidious onset of arthralgias one to two weeks after the initial ER visit with subjective fevers and persistent rash; all the symptoms were getting worse. The patient later returned to the ER on day 62 with complaints of flu-like symptoms, intense joint pains, intermittent subjective fever, and chills with a persistent rash on the chest. The patient was found to have a febrile temperature of 38.7°C in triage with tachycardia of 107 beats per minute (bpm). His lab reports were significant for a positive throat swab for GAS, a white blood cell (WBC) count of 11,000 cells/μL, AST of 43 IU/L, and ALT of 87 IU/L. His chest X-ray was negative for acute pathology. The patient was treated with clindamycin 900 mg IV and discharged from the ER with a diagnosis of GAS throat infection and was prescribed a short course of oral clindamycin.

**Figure 1:**
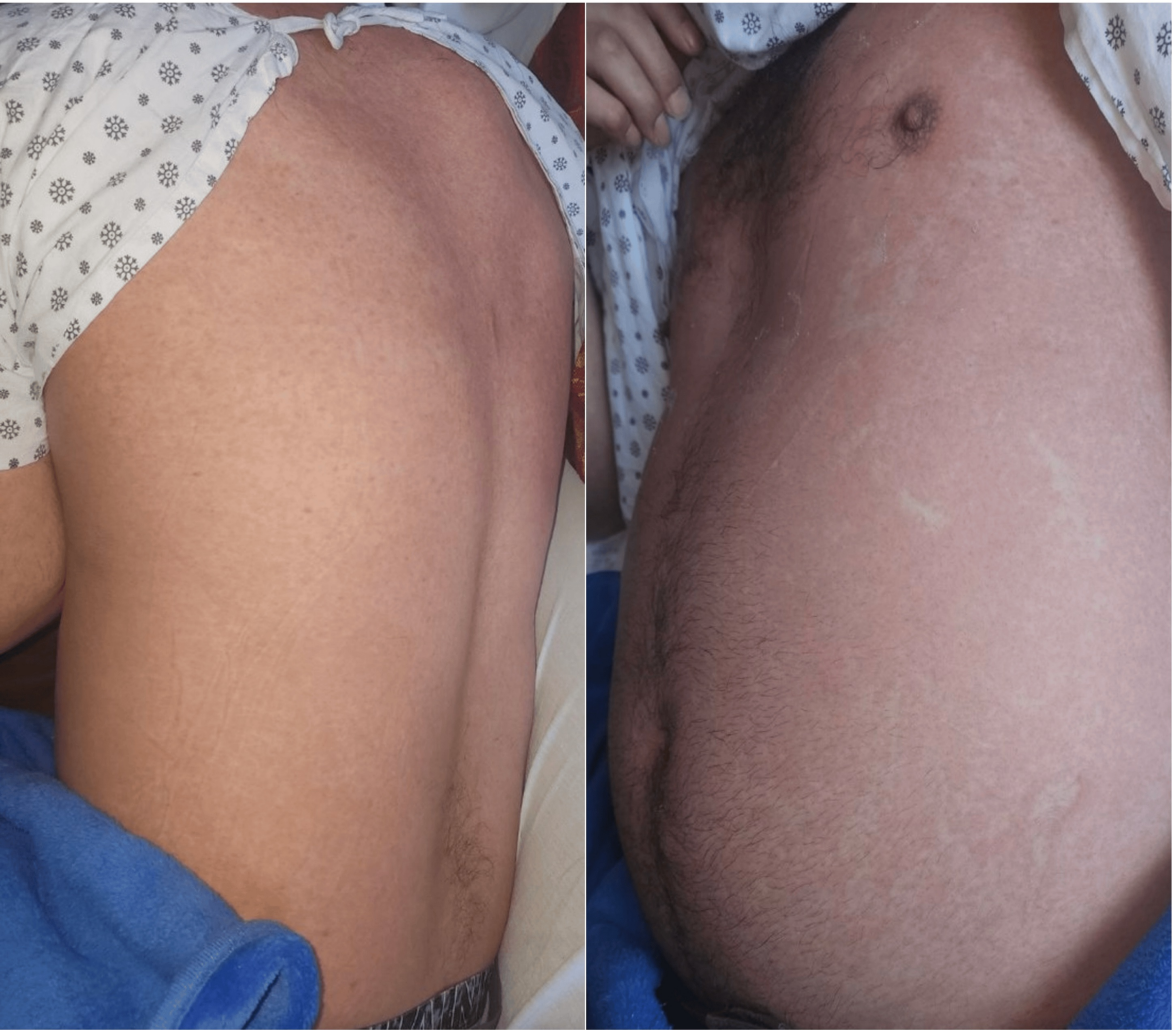
Salmon-colored rash noted on the patient

The patient returned to the ER a few days later, on day 69, with persistent but worsening sore throat, muscle aches, joint stiffness, and intermittent fever. He was febrile in the ER with a temperature of 38.8 °C and tachycardia of 112 bpm. The patient denied any recent travels or hiking. His labs were significant for elevated WBC of 19,900 cells/μL, AST of 58 IU/L, ALT of 149 IU/L, nonreactive HIV antigen-antibody, lactic acidosis of 4.5 mmol/L, CRP of 232 mg/L, ESR of 96 mm/hr. Sepsis protocol was initiated, and the patient received weight-based fluid resuscitation, received intramuscular penicillin G injection 1.2 million units with a stat dose of vancomycin 1,500 mg, with minimal symptomatic relief. A CT scan of the neck with IV contrast was normal; the patient was discharged from the ED the same day and recommended to follow up with a primary care provider (PCP). However, the patient returned to the ED on day 72 with a worsening rash (Figure 1).

The patient denied any sick contacts and was admitted to the medicine service with suspicion of acute rheumatic fever (ARF) secondary to GAS. The patient was started on empirical antibiotic coverage with vancomycin and piperacillin-sulbactam for GAS infection treatment; on the fourth visit, admission, labs were significant for persistent leucocytosis and further worsening. Reports of AST of 460 IU/L/ALT of 847 IU/L and ultrasound of the abdomen showed diffuse hepatic steatosis with a nonreactive viral hepatitis panel for hepatitis A, B, and C. During the initial days of admission, the patient complained of migratory arthralgia in the bilateral wrist, right shoulder, right knee, and left ankle with redness or swelling of affected joints. X-ray imaging of affected joints showed no acute pathology; a CT scan of the chest, abdomen, and pelvis done with contrast was notable for bilateral atelectasis. no hepatosplenomegaly or lymphadenopathy were noted. Antistreptolysin-O and anti-Dnase titles were found to be in normal ranges.

**Figure 2:**
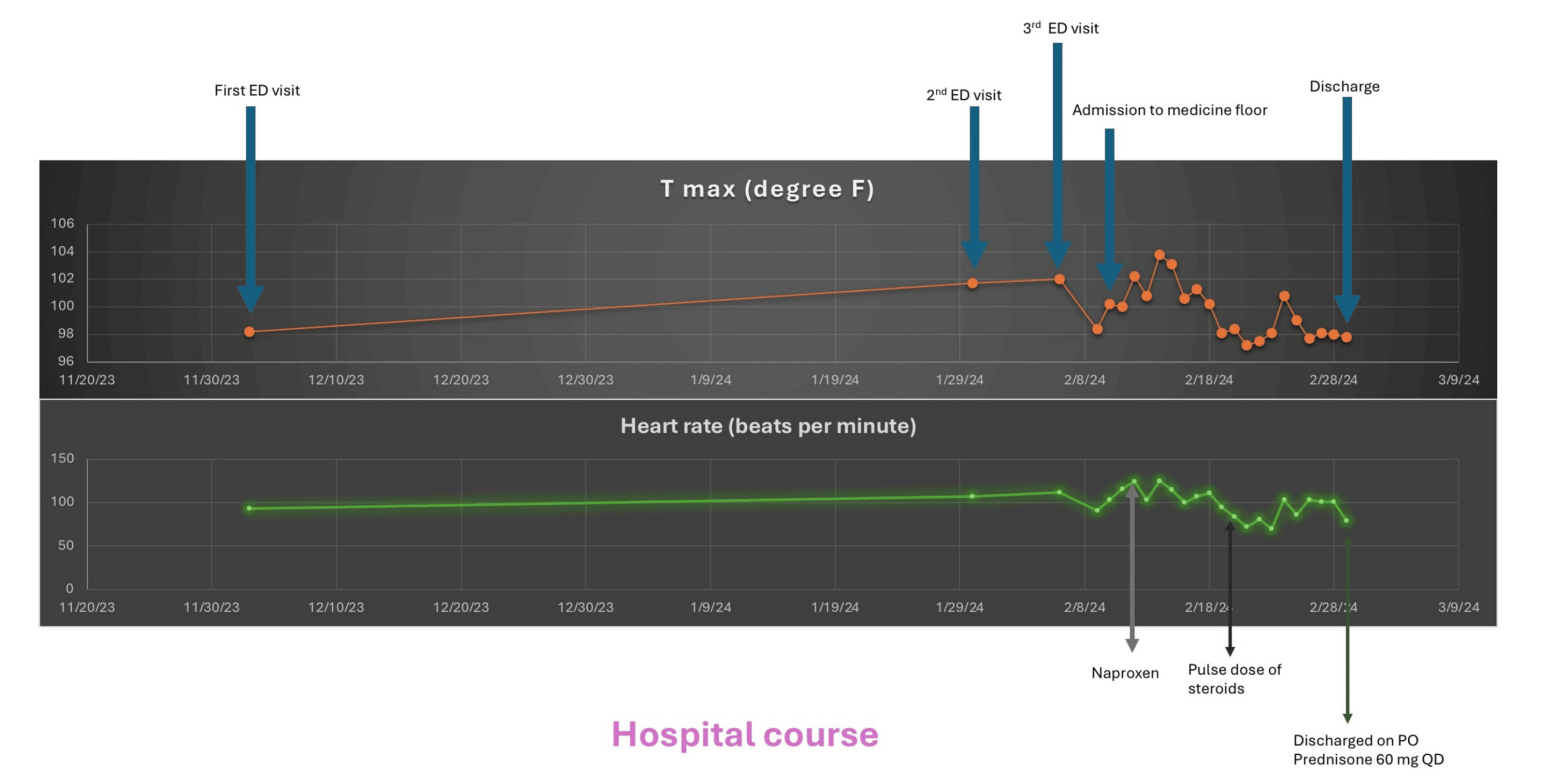
Clinical course including maximum recorded temperature and heart rate PO: per oral; QD: four times a day

We considered several differential diagnoses, focusing mainly on infection mimics such as ARF due to the overlap in symptoms with AOSD. Other mimics, like infections, including viral as well as bacterial, were ruled out as per infectious diseases consult. Hematology was consulted and recommended that his symptoms and labs were consistent with inflammatory syndrome rather than malignancy. On day 75, naproxen 500 mg twice daily was started; during the days following appropriate non-steroidal anti-inflammatory drug (NSAID) dosing, arthralgia resolved on day 78. The patient had symptomatic relief; however, he did have a persistent rash and worsening liver function and was found to have hyperferritinemia of 88,000 ng/mL and an IL-2 level of 13,277 pg/mL. The patient met the Yamaguchi criteria for AOSD, including arthralgia for more than two weeks, typical rash, a WBC count of more than 10,000 cells/μL, sore throat, and abnormal liver enzymes, with GAS being the suspected triggering factor. Figure 2 demonstrates the vitals during the inpatient stay.

**Figure 3:**
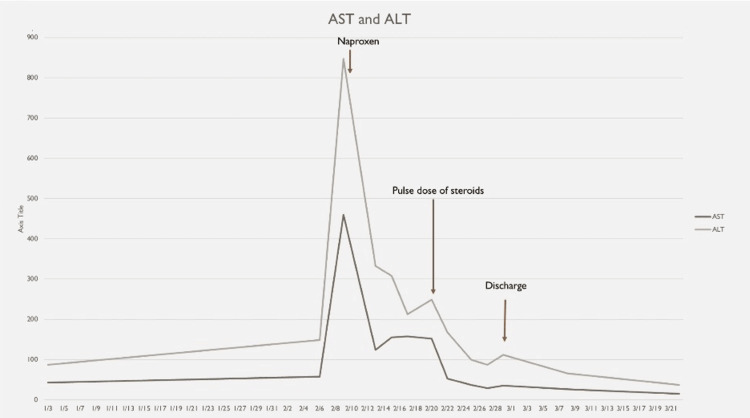
The trend of AST and ALT during the hospital stay; an initial downtrend was seen upon starting NSAIDs, with a further downtrend after a pulse dose of steroids, and both AST and ALT levels trended down to normal ranges upon discharge. AST: aspartate aminotransferase; ALT: alanine aminotransferase; NSAID: non-steroidal anti-inflammatory drug

**Figure 4:**
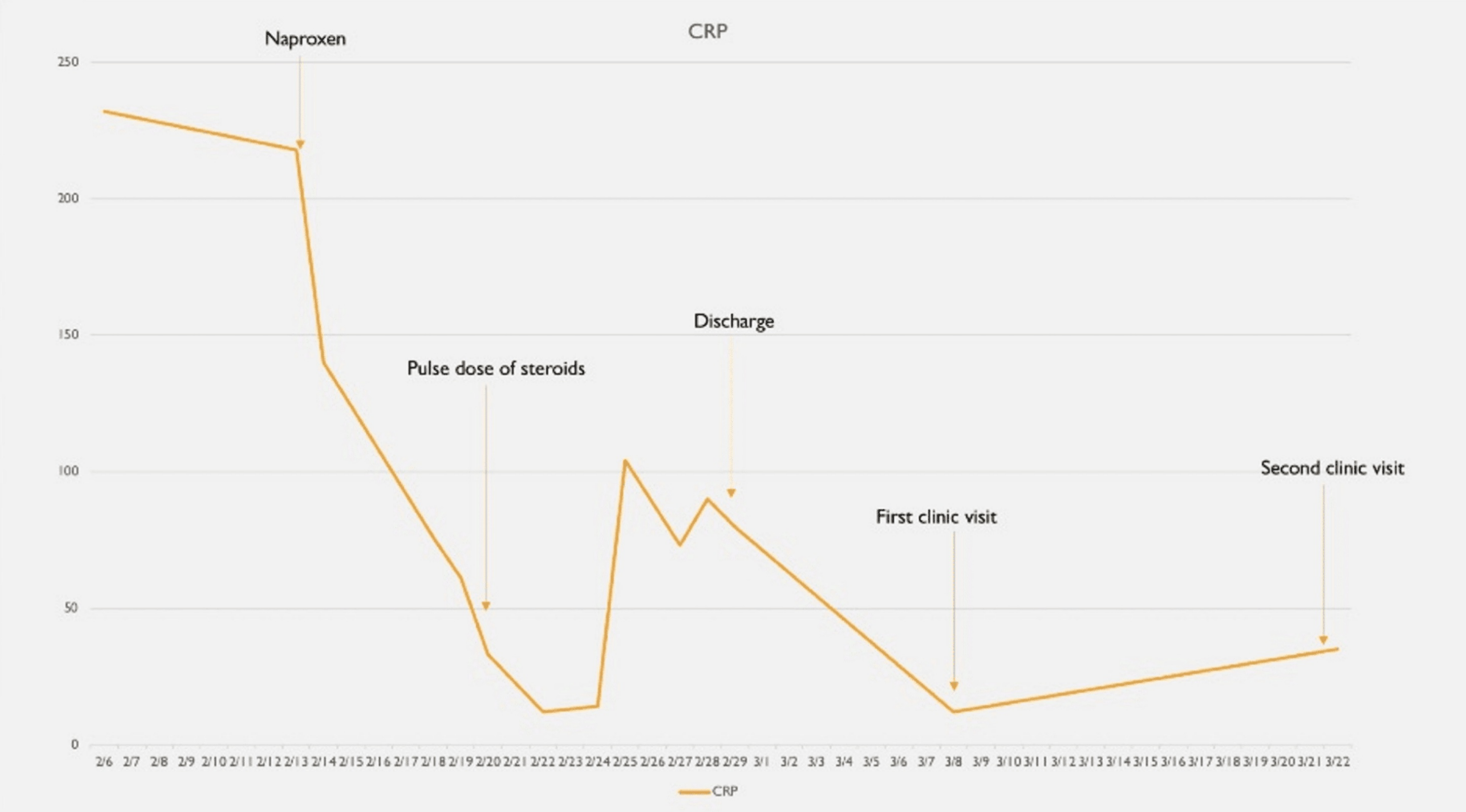
The trend of CRP during hospital stay; an initial downtrend was seen upon starting NSAIDs, with a further downtrend after the pulse dose of steroids, and levels further trended down upon discharge. CRP: C-reactive protein; NSAID: non-steroidal anti-inflammatory drug

**Figure 5:**
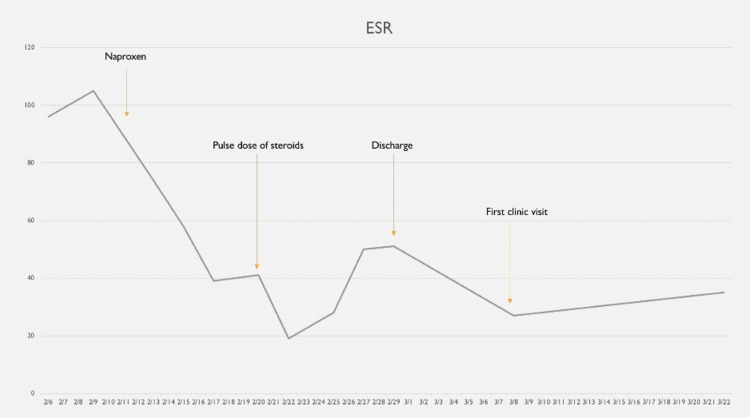
The trend of ESR during the hospital stay; an initial downtrend was seen upon starting NSAIDs, with a further downtrend after the pulse dose of steroids, and levels further trended down upon discharge ESR: erythrocyte sedimentation rate; NSAID: non-steroidal anti-inflammatory drug

**Figure 6:**
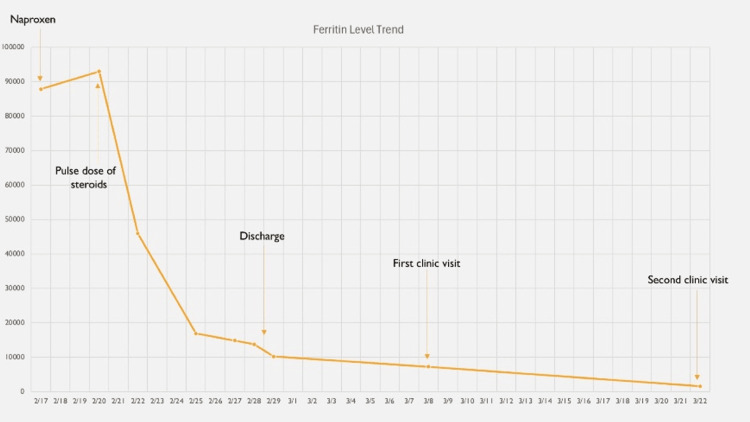
The trend of ferritin levels during hospital stay; an initial downtrend was seen upon starting the pulse dose of steroids, and levels further trended down upon discharge.

Clinical exam and hyperferritinemia of 92,999 ng/mL were suggestive of severe systemic inflammation and organ involvement-liver, associated with AOSD, hence, three days of methylprednisone 1,000 mg was given from day 83 till day 85, resulting in quick resolution of symptoms, followed by slow tapering, high dose steroids during the inpatient stay. One mg/kg oral prednisone was used due to the persistence of severe symptoms or organ involvement, significantly elevated inflammatory markers as listed in Table 1, requiring more aggressive immunosuppression, following which fever episodes resolved with a downtrend of ferritin levels, CRP, ESR, and improving AST/ALT levels (Figures 3-6).

**Table 1:** Laboratory investigations WBC: white blood count; NA: not available; GAS: Group A *Streptococcus*; ESR: erythrocyte sedimentation rate; CRP: C-reactive protein; ALP: alkaline phosphate; AST: aspartate transaminase; ALT: alanine transaminase; PCR: polymerase chain reaction; IL-2: interleukin-2; INR: international normalised ratio PTT: partial thromboplastin time; INR: international normalized ratio; CMV: cytomegalovirus; EBV: Epstein-Barr virus; ACE: angiotensin-converting enzyme; ALL panel: acute lymphocytic leukemia

Parameter	3 January	6 February	9 February	13 February	15 February	17 February	20 February	22 February	25 February	27 February	29 February	8 March	22 March
WBC (4000-11000/microliter)	11.8	19.9	19.7	18.6	15.4	9.68	22.4	14.4	15.7	14.4	20.6	16.5	10.2
Hemoglobin (13.8-17.2 g/dL)	14.3	14.5	14.6	11.8	11.9	11.8	12.4	11.9	12	11.7	12	12.7	13.3
Platelets (150-450 K/UL)	438	326	358	318	300	203	238	331	433	279	265	255	258
GAS swab	Detected	NA	NA	NA	NA	NA	NA	NA	NA	NA	NA	NA	NA
ESR (0-15 mm/hr)	NA	96	105	74	58	39	41	19	28	50	51	27	35
CRP (<1 mg/L)	NA	232	NA	218	124	76.3	33.3	12.2	104	73.3	80.2	12.3	35.9
Ferritin (24-336 ng/l)	NA	NA	NA	NA	NA	87855	92999	45904	16923	14854	10255	7275	1568
Procalcitonin (<1 ng/mL)	0.31	0.56	NA	NA	1.37	NA	1.31	NA	0.24	NA	NA	NA	NA
ALP (44-147 IU/L)	123	177	371	288	382	362	370	291	201	183	192	138	88
AST (8-48 IU/L)	43	58	460	124	155	158	152	53	37	29	36	27	15
ALT (7-55 IU/L)	87	149	847	333	308	213	249	168	100	87	112	66	37
Blood culture	No growth	No growth	No growth	No growth	NA	NA	NA	NA	No growth	NA	NA	NA	NA
SARS-CoV-2 PCR	Negative	Negative		NA	NA	Positive	Negative	NA	Negative	NA	NA	NA	NA
IL-2 (0- 31.2 pg/mL)	NA	NA	NA	NA	NA	NA	13277	NA	NA	NA	NA	NA	NA
INR (0.8-1.1)	NA	NA	NA	NA	1.26	NA	1.01	NA	NA	NA	NA	NA	NA
PTT (25-35 s)	NA	NA	NA	NA	31.2	NA	23.5	NA	NA	NA	NA	NA	NA
Qunatiferon	NA	NA	NA	NA	NA	NA	Negative	NA	NA	NA	NA	NA	NA
HIV	NA	Negative	NA	NA	Negative	NA	NA	NA	NA	NA	NA	NA	NA
Hepatitis B surface antigen	NA	NA	NA	NA	Non reactive	NA	NA	NA	NA	NA	NA	NA	NA
Hepatitis C antibody	NA	NA	NA	NA	Non reactive	NA	NA	NA	NA	NA	NA	NA	NA
CMV	NA	NA	NA	NA	Not detected	NA	NA	NA	NA	NA	NA	NA	NA
EBV	NA	NA	NA	NA	Not detected	NA	NA	NA	NA	NA	NA	NA	NA
Parvovirus	NA	NA	NA	NA	Not detected	NA	NA	NA	NA	NA	NA	NA	NA
*Brucella *IgG, IgM (<0.80)	NA	NA	NA	NA	0.16, 0.05	NA	NA	NA	NA	NA	NA	NA	NA
*Bartonella *IgG	NA	NA	NA	NA	Not detected	NA	NA	NA	NA	NA	NA	NA	NA
*Leptospira *DNA quantification	NA	NA	NA	NA	Not detected	NA	NA	NA	NA	NA	NA	NA	NA
Antistreptolysin antibody (<166 Todd units)	NA	NA	NA	128	NA	NA	NA	139	116	NA	NA	87	NA
Anti- DNase B (0-302 U/mL)	NA	NA	NA	192	NA	NA	NA	NA	NA	NA	NA	208	NA
Chlamydia trachomatis	NA	NA	NA	Not detected	Not detected	NA	NA	NA	NA	NA	NA	NA	NA
Neisseria gonorrhea	NA	NA	NA	Not detected	Not detected	NA	NA	NA	NA	NA	NA	NA	NA
Syphilis *Treponema pallidum*	NA	NA	NA	NA	Negative	NA	NA	NA	NA	NA	NA	NA	NA
*Toxoplasma *IgM (< 8 AU/mL)	NA	NA	NA	<8.00	NA	NA	NA	NA	NA	NA	NA	NA	NA
ACE level (<40 microgram/L)	NA	NA	NA	NA	NA	70	NA	NA	NA	NA	NA	NA	NA
ALL panel	NA	NA	NA	NA	NA	NA	Negative	NA	NA	NA	NA	NA	NA
Babesiosis IgM (< 1:20)	NA	NA	NA	NA	NA	NA	< 1:20	NA	NA	NA	NA	NA	NA
*Anaplasma phagocytophilum* IgM (< 1:16)	NA	NA	NA	NA	NA	NA	< 1:20	NA	NA	NA	NA	NA	NA
*Ehrlichia chaffeensis* IgM (< 1:20)	NA	NA	NA	NA	NA	NA	< 1:20	NA	NA	NA	NA	NA	NA

A skin biopsy was done, and it showed perivascular dermatitis with extravasated erythrocytes and rare eosinophils and neutrophils. The patient was discharged on day 91 after an extended inpatient stay on oral prednisone 60 mg daily. The patient was seen in an outpatient setting on day 99 and found to have a resolving rash on physical examination and a further downtrend in inflammatory markers; the patient was continued on prednisone 60 mg four times a day (QD). Following the clinic visit on day 114, the patient was tolerating slow tapering of steroids and was noted to have decreased left knee effusion; however, the patient reported bilateral knee pain and left elbow pain. The patient was started on methotrexate 7.5 mg and prednisone was tapered down to 50 mg QD. Methotrexate is typically initiated at 15-25 mg/week in autoimmune diseases like AOSD. We started with 7.5 mg as there were concerns about liver function (elevated liver enzymes) but it was increased to 15 mg/week on the next follow-up visit after blood showed normalization of liver function. We tapered steroids along with starting methotrexate, hence, to achieve disease control with the tapering and eventual discontinuation of steroids, we transitioned the patient to steroid-sparing agent methotrexate. Table 1 demonstrates various laboratory investigations done for the patient.

## Discussion

Adult-onset Still's disease is a rare clinical entity. Due to the lack of definitive diagnostic tests, diagnosing AOSD is challenging, and clinical criteria hold an essential place in diagnosing this life-threatening condition. Yamaguchi criteria is widely used in diagnosing AOSD; major criteria include fever of at least 39°C for at least one week, arthralgia or arthritis for at least two weeks, non-pruritic salmon-colored rash on the trunk/extremities, granulocytic leukocytosis (10,000/mL or greater), and minor criteria are sore throat, lymphadenopathy, hepatomegaly or splenomegaly, abnormal liver function tests, and negative in tests for rheumatoid factor (RF) and ANA. [10], Lebrun et al. retrospectively reviewed the validity of diagnostic criteria for AOSD and published that Yamaguchi criteria are 92.1% sensitive and 98.5% specific [11]. Abiding by clinical diagnostic criteria helps yield an early diagnosis. In 2001, Fautrel et al. studied the diagnostic value of ferritin level in AOSD, and it was found that a combination of serum ferritin levels five times the usual and glycosylated ferritin levels less than or equal to 20% yielded 92.9% specificity [12].

Though our patient had three ED visits for on and off subjective fevers, sore throat, non-resolving rash, and worsening migratory polyarthralgia, he was admitted to the medicine floor after eight weeks of initial clinical presentation, therefore, having high clinical suspicion is crucial to prevent delayed or misdiagnosis. Presentation of AOSD is often found to be very similar to other systemic diseases [13]; our patient was initially admitted to service for suspected ARF as the patient met Jones criteria as well, with one major (polyarthritis) and two minor criteria (fever, arthralgia, elevated inflammatory markers, leucocytosis). Later laboratory tests, including anti-DNase and anti-streptolysin O titles, were nonindicative of ARF. Echocardiography ruled out any valvular disease. In 2014, Karagoz et al. published a case report similar to ours, where AOSD was found to mimic ARF, and in both cases, significantly higher ferritin levels and a reasonable response to steroids helped exclude other infections or malignancies [14].

The proposed mechanism for AOSD is similar to other autoimmune diseases, likely secondary to various triggers in genetically predisposed individuals [15]. Borg et al. revealed a case of a 78-year-old male with AOSD that was preceded by an Epstein-Barr virus (EBV) infection detected by polymerase chain reaction (PCR) [16]. Sharabi et al., in 2021, reported two cases of COVID-19 vaccination-induced hyper-inflammatory response resulting in AOSD [17]. Fossil et al. reported an interesting case of AOSD following reactivation of human herpesvirus 6 (HHV-6) viral infection in an immunocompetent 78-year-old male postulating viral infection triggered dysregulation of the immune system [18]. In 2022, AlQudari et al. presented a rare case of possibly vaccination-triggered AOSD after COVID-19 vaccination in a 29-year-old male, highlighting the importance of figuring out etiologies associated with autoimmune activation [19].

Given widespread microbial and non-microbial triggers, there is growing evidence that infections could cause these rare disorders. Due to the growing incidence of these infections, investing resources to study the agents inducing inflammatory responses resulting in autoimmunity is appropriate. Although it is well known that GAS pharyngitis patients should receive prophylactic antibiotics to prevent ARF and eradicate GAS carriage, patients who have had ARF benefit from long-term antibiotics in terms of preventing recurrences and severity of ARF in subsequent attacks [20]. Our patient is suspected to have developed AOSD after a GAS infection. We suspect the infection triggered an inflammatory cascade, resulting in a heightened inflammatory response. It is unclear whether secondary antibiotic prophylaxis would prevent subsequent recurrences and severity of future attacks of AOSD. Detailed clinical studies are needed to provide clarity, as AOSD itself carries significant mortality and morbidity.

## Conclusions

Our case report highlights the difficulty with diagnosing AOSD. As this is a clinical diagnosis, the most commonly used is the Yamaguchi criteria. However, these criteria have high sensitivity but very low specificity, so high clinician suspicion aided by diagnostic criteria and blood work can help diagnose these inflammatory syndromes. Along with diagnosing the disease, understanding the etiology would push clinicians to suspect these rare pathologies. Multiple authors have reported cases in the literature to identify various triggering factors. The case we are reporting is to isolate GAS as one of the etiologies for AOSD, although further epidemiological studies are needed to quantify the genuine associated risk with these triggers.

## References

[1] Leavis HL, van Daele PL, Mulders-Manders C (2024). Management of adult-onset Still's disease: evidence- and consensus-based recommendations by experts. Rheumatology (Oxford).

[2] Awoyemi T, Conti A, Aguilar FG (2023). Adult-onset Still's disease complicated by macrophage activation syndrome. Clin Case Rep.

[3] Yaman F, Kimiaei A (2023). A case of adult-onset Still's disease in a patient after a car accident. Clin Case Rep.

[4] Bhargava J, Panginikkod S (2024). Still Disease. https://www.ncbi.nlm.nih.gov/books/NBK538345/.

[5] Komiya A, Matsui T, Nogi S (2012). Neutrophil CD64 is upregulated in patients with active adult-onset Still's disease. Scand J Rheumatol.

[6] Chen DY, Lan JL, Lin FJ, Hsieh TY, Wen MC (2004). Predominance of Th1 cytokine in peripheral blood and pathological tissues of patients with active untreated adult onset Still's disease. Ann Rheum Dis.

[7] Shimizu M, Takei S, Mori M, Yachie A (2022). Pathogenic roles and diagnostic utility of interleukin-18 in autoinflammatory diseases. Front Immunol.

[8] Cota-Arce JM, Cota J, De León-Nava MA (2021). Efficacy and safety of canakinumab in the treatment of adult-onset Still's disease: a systematic review. Semin Arthritis Rheum.

[9] Choi JH, Suh CH, Lee YM (2003). Serum cytokine profiles in patients with adult onset Still's disease. J Rheumatol.

[10] Yang JW, Lee E, Seo JY, Jung JY, Suh CH, Kim HA (2018). Application of the international league against rheumatism classification criteria for systemic juvenile idiopathic arthritis as a prognostic factor in patients with adults-onset Still's disease. Pediatr Rheumatol Online J.

[11] Lebrun D, Mestrallet S, Dehoux M (2018). Validation of the Fautrel classification criteria for adult-onset Still's disease. Semin Arthritis Rheum.

[12] Fautrel B, Le Moël G, Saint-Marcoux B (2001). Diagnostic value of ferritin and glycosylated ferritin in adult onset Still's disease. J Rheumatol.

[13] Mankgele M, Hlawe D, Fabiano Z (2023). An initially missed diagnosis of venous thromboembolic phenomenon in adult-onset Still’s disease: a case report and literature review. Am J Case Rep.

[14] Karagoz E, Ozalper V, Ulcay A, Turhan V, Top C (2014). Adult-onset still's disease mimicking acute rheumatic fever. Turk J Phys Med Rehabil.

[15] Kobayashi T, Hashimoto K, Kusanagi Y, Tanaka Y (2023). Occurrence of adult-onset Still's disease after coronavirus disease 2019 BNT162B2 vaccination in a patient with ulcerative colitis: a case report and review of literature. Clin Case Rep.

[16] Borg J, Camilleri ML, Cassar PJ (2020). Adult-onset Still's disease in a 73-year-old Maltese man. BMJ Case Rep.

[17] Sharabi A, Shiber S, Molad Y (2021). Adult-onset Still's disease following mRNA COVID-19 vaccination. Clin Immunol.

[18] Rossio R, Colombo G, Piconi S, Peyvandi F (2021). Adult-onset still disease after human herpesvirus 6 infection in an elderly patient: a case report. J Clin Rheumatol.

[19] AlQudari EA, Alabdan LI, Alkhathami AA, Alotaibi MD, Alhamzi HA (2022). Adult-onset Still’s disease after the ChAdOx1 nCoV-19 vaccine. Cureus.

[20] Al-Jazairi A, Al-Jaser R, Al-Halees Z (2017). Guidelines for the secondary prevention of rheumatic heart disease: endorsed by Saudi Pediatric Infectious Diseases Society (SPIDS). Int J Pediatr Adolesc Med.

